# Revisiting sulfur H-bonds in proteins: The example of peroxiredoxin AhpE

**DOI:** 10.1038/srep30369

**Published:** 2016-07-29

**Authors:** Laura A. H. van Bergen, Mercedes Alonso, Anna Palló, Lennart Nilsson, Frank De Proft, Joris Messens

**Affiliations:** 1 Research Group of General Chemistry, Vrije Universiteit Brussel, 1050 Brussels, Belgium; 2 Structural Biology Research Center, VIB, 1050 Brussels, Belgium; 3 Brussels Center for Redox Biology, 1050 Brussels, Belgium; 4 Structural Biology Brussels, Vrije Universiteit Brussel, 1050 Brussels, Belgium; 5 Department of Biosciences and Nutrition, Karolinska Institutet, SE-14183 Huddinge, Sweden

## Abstract

In many established methods, identification of hydrogen bonds (H-bonds) is primarily based on pairwise comparison of distances between atoms. These methods often give rise to systematic errors when sulfur is involved. A more accurate method is the non-covalent interaction index, which determines the strength of the H-bonds based on the associated electron density and its gradient. We applied the NCI index on the active site of a single-cysteine peroxiredoxin. We found a different sulfur hydrogen-bonding network to that typically found by established methods, and we propose a more accurate equation for determining sulfur H-bonds based on geometrical criteria. This new algorithm will be implemented in the next release of the widely-used CHARMM program (version 41b), and will be particularly useful for analyzing water molecule-mediated H-bonds involving different atom types. Furthermore, based on the identification of the weakest sulfur-water H-bond, the location of hydrogen peroxide for the nucleophilic attack by the cysteine sulfur can be predicted. In general, current methods to determine H-bonds will need to be reevaluated, thereby leading to better understanding of the catalytic mechanisms in which sulfur chemistry is involved.

Most hydrogen bonds (H-bonds) focus on oxygen and nitrogen atoms as both acceptors and donors, with less attention devoted to sulfur, which is also known to form H-bonds, and whose nucleophilic character is essential for numerous enzymatic reactions^1^. As a consequence, and due to the historical focus on nitrogen and oxygen, the definition of a H-bond is primary based on the characteristics of oxygen and nitrogen^2^. Whether a hydrogen bond is formed or not depends on the electronegativity of an atom, as electronegativity is the power of an atom to attract electrons to itself.

To locate H-bonds in proteins, various algorithms can be used^3^,^4^,^5^,^6^,^7^ corresponding to either geometrical or so-called pseudo-energetic criteria. Most frequently, the geometrical criteria are used to find H-bonds in proteins by measuring the maximum distance between acceptor and donor (D
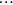
A) or the maximum distance between hydrogen and acceptor (H
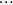
A). In several codes^6^, the distances are complemented with an additional maximum angle divergence, starting from a perfect linear 180° donor—hydrogen—acceptor (D-H
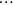
A) angle, to a user-dependent cutoff value. However, H-bonds are mainly electrostatic in nature and therefore interactions over distances larger than the van der Waals radii cutoffs are possible^8^.

Previous research on protein cysteine sulfurs showed that the geometric characteristics of H-bonds involving sulfur atoms are clearly different from those involving oxygen and nitrogen atoms^9^, and so the use of equivalent structural parameters often results in incorrect and/or incomplete interpretations. Although the average H-bond length of a cysteine acting as a donor is 2.51 Å (S-H
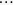
X), it can increase up to 2.84 Å depending on the nature of the acceptor^9^, while the cutoff for identifying H-bonds is usually set at a distance of 2.40 Å. An even more notable deviation from the conventional donor-acceptor distance is that a cysteine sulfur as acceptor has an average H-bond length of 2.80 Å (X-H
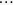
S), which is likely to remain unnoticed by conventional programs due to the large deviation from the standard H
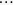
A distance of 2.40 Å^9^. Very recently, it was also shown that the amide N-H
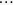
S H-bonds in biomolecules are equally strong H-bonds as their oxygen counterpart, confirming that the electronegativities of the acceptors cannot be the sole criteria to predict the H-bond strength^10^.

Here, we have compared several established methods for detecting H-bonds in proteins. These methods use different types of geometrical or pseudo-energetic criteria. We assessed the performance of these methods in the identification of sulfur hydrogen bonds within the active site of the enzyme Alkyl hydroperoxide reductase E (AhpE) from *Mycobacterium tuberculosis*. We found that the conventional methods for H-bond detection cannot detect all of the H-bonds that are important for the understanding of the reaction mechanism of AhpE involving the cysteine sulfur of the active site. By applying the Non-Covalent Interaction (NCI) method^11^ previously used to reveal non-covalent interactions in molecular systems and solids^12^,^13^,^14^, and by taking into account the strength of each individual sulfur H-bonds, we show that sulfur H-bonding can take place over longer distances than the standard conventional H-bonds, and with strength comparable to that of the conventional shorter nitrogen and oxygen H-bonds. Accordingly, the cutoff distance for H-bonds should be based on the type of heavy atoms involved, and therefore we propose a new geometry-based algorithm for identification of all types of H-bonds in proteins, which will be implemented in the next release of CHARMM (version 41b), whose classical force fields were previously scrutinized for simulation studies of structurally related sulfur-containing proteins^15^,^16^.

## Results

In order to perform a critical assessment of conventional and NCI methods for detecting H-bonds involving the peroxidatic sulfur atom in AhpE, we proceeded as follows. First, the crystal structure of the reduced AhpE structure was re-refined based upon findings during the MD simulation of the original structure (1xxu). The crystal structure of AhpE shows 2 dimers per asymmetric unit, which are denoted chains A and B^17^. Accordingly, we decided to use both chains in our analysis, because the cysteine environment in each is distinctly different. In chain A, the side chain of the conserved arginine residue (Arg116) is oriented away from the cysteine and directed outwards to the flexible β9-α5 loop, whilst in chain B it is turned inwards to the active site cysteine (Fig. S1). The two chains of the re-refined structure (4x0x) were heated to 300 K and equilibrated using MD simulations, and three different conventional methods based on geometrical or pseudo-energetic criteria were used to detect the H-bonds involving the peroxidatic Cys45 both as thiolate and thiol. Additionally, the recently developed NCI index was also used to establish unambiguously the H-bonding network of the AhpE structure. Finally, the strength of individual sulfur H-bonds provided by the NCI index were used to predict ligand docking in the active site, and then also applied in the design of an improved equation to identify H-bonds in proteins through use of an adapted distance criterion taking the chemical nature of the atoms into account.

### Refining the structure of AhpE

**Figure 1:**
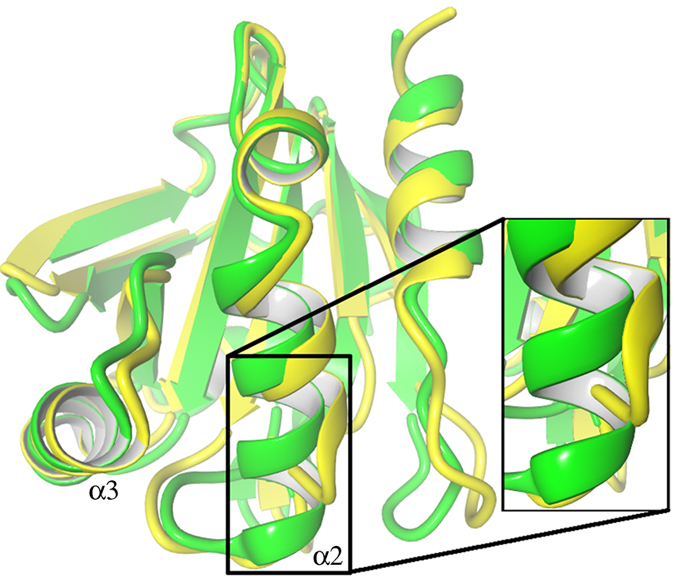
AhpE protein instability during MD simulations. The opening between α2 and α3 helices induced by 30 ns of simulation in the deposited original chain A structure (PDB 1 xxu) (yellow), with comparison to the more stable re-refined structure (PDB 4x0x) (green). The original structure of AhpE starts to unwind at the end of the α2 helix, in which Cys45 is located. The re-refined structure (green) does not unwind in this same time frame.

During the MD simulation with reduced AhpE (PDB: 1xxu), we found that between the 20^th^ and 25^th^ ps, the glutamine located next to the cysteine (Gln46) flips its side chain during the heating phase of chain A, resulting in a change of dihedral χ_3_ from +150° to −90°. This switching of the positions of the Oε1 and Nε2 of Gln46 leads to helix α2 moving away from helix α3, creating an empty space of ~20 Å^3^. After performing a 30 ns simulation, these helices move even further apart, and the first turn of α2, where Cys45 is located, completely unwinds (Fig. 1). To improve protein stability during MD simulations, we realized that a re-refinement of the models of the AhpE structure was needed (see supplementary). After this re-refinement operation (Fig. S2), we repeated the simulation with the newly refined structure for chain A and found that after 30 ns the helix α2 remained intact (Fig. 1). The new structural models of AhpE (4x0x) represent the experimental data with an average improvement of 3% in the attributed R-factors (Table S1). Importantly, a *gauche* minus conformation was found for the peroxydatic Cys45 in the active site of chain A after re-refinement, which is in line with the conformation reported for most of peroxiredoxins (Fig. S2).

### Identification of sulfur hydrogen bonds using conventional methods

We tested three conventional methods for determining H-bonds in chains A and B of the re-refined AhpE. These methods assume that all types of H-bonds interact over the same range of distances and angles. Method 1 uses the H
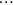
A distance as the geometrical indicator, which is set to 2.4 Å by default. This method needs input from the user, who decides which atoms act as donors or acceptors. Method 2 uses both distance cutoff (D
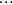
A) and angle cutoff (D-H···A) standards of 3.0 Å and 160°–180°, respectively. Method 3 uses a pseudo-energetic expression^7^, which is based on distances and angles (See supplementary for details). This method only assigns a single H-bond to every hydrogen atom in the structure, and therefore, bifurcated H-bonds go unnoticed.

**Figure 2:**
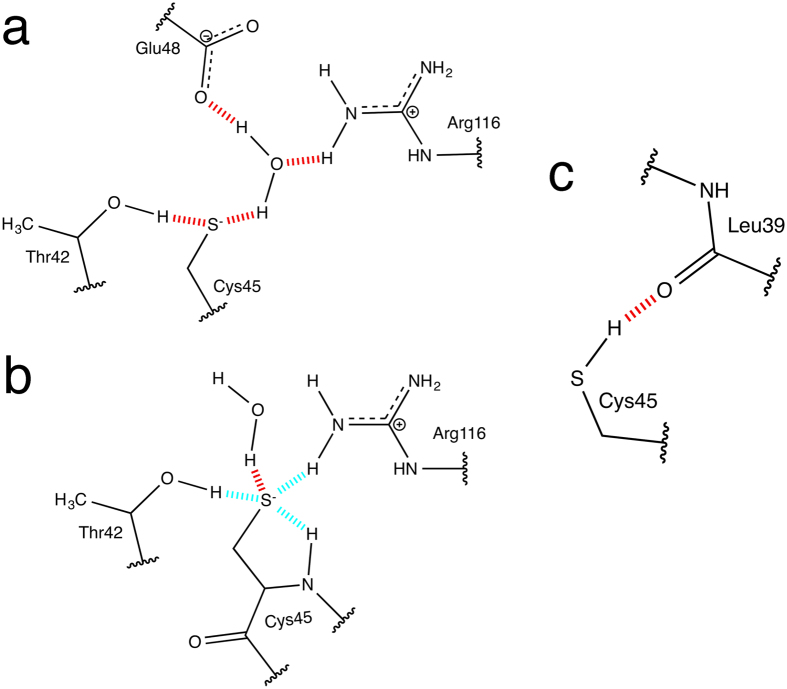
The H-bond networks found by the conventional methods. Red colored H-bonds are found with Method 1 H
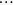
A cutoff of 2.4 Å for all-atoms) and Method 3. Blue colored H-bonds are only found with Method 1. (**a)** The H-bond network of chain A with Cys45 modeled as a thiolate. (**b**) The H-bond network of chain B with Cys45 modeled as a thiolate. (**c**) The H-bond network when Cys45 modeled as a thiol for both chain A and B.

The three methods were tested on the equilibrated chain A and B of AhpE with the peroxidatic Cys45 once as thiolate and once as thiol. With Cys45 as thiolate, Method 2 resulted in no H-bonding with the Cys45 sulfur, and this for both chains (Fig. 2a). Methods 1 and 3 gave a similar H-bonding network for Cys45 in chain A (Fig. 2a). Here, the side chain of Thr42 directly donates a H-bond to the Cys45 sulfur, and a water molecule bridges Cys45 with both Arg116 and Glu48. For chain B (Fig. 2b), however, Methods 1 and 3 result in a different H-bonding network. Method 1 finds four H-bonds; one in Cys45 interacting with its own backbone, one with the side chain of Thr42, one with the side chain of Arg116, and one with water. Method 3, however, only finds the interaction with the water molecule, not the Arg116. It is predicted that stabilization of the Cys45 thiolate by Arg116 will promote its nucleophilic character, so that this Cys represents a catalytically active state of the enzyme^18^,^19^. Successful identification of this interaction is therefore vital for understanding the active catalytic form of this peroxiredoxin.

Next, we applied all three methods on chain A and B with Cys45 as thiol. Also here for the Methods 1 and 3 (Fig. 2c) the same result was found, Cys45 donates a H-bond to the backbone oxygen of Leu39, while Method 2 finds no H-bonds.

Important to note is that the H-bond cutoff settings used in Method 1 can be easily changed by the user. Therefore, we tested the cutoff at 3.0 Å for H-bonds involving the sulfur atom, while keeping the maximum H-bond distance to the standard 2.4 Å for all other H-bonds (Fig. S7). In this case, for Chain A with Cys45 as thiolate, three extra sulfur H-bonds were found, and for Chain B, one additional sulfur H-bond with Arg116 was found.

All in all, applying these conventional methods results in a different outcome, which strongly depends on the user-defined geometrical cutoffs.

### More H-bonds with the Non-Covalent Interaction (NCI) index

A recently developed method to reveal non-covalent interactions in real space is the Non-Covalent Interaction (NCI) index^11^. In particular, the NCI approach allows for characterization of non-covalent interactions in 3D space based on peaks that appear in the reduced density gradient at low densities. Regions where the electron density and the reduced density gradient are low correspond to regions where non-covalent interactions occur. Importantly, the strength of each interaction is derived from the density values of the low-gradient spikes. Dispersion interactions usually appear at very low densities (*ρ* < 0.01 a.u.)^13^ whereas stronger H-bonds appear at higher density values (0.01 < *ρ* < 0.06 a.u.)^20^. To distinguish between attractive and repulsive interactions, the NCI index uses the sign of the second eigenvalue of the electron-density Hessian matrix (*λ*_2_), in such a way that *λ*_2_ < 0 indicates bonding interactions, whereas *λ*_2_ > 0 point out repulsive interactions^11^. Accordingly, the reduced density gradient is usually plotted against sign(*λ*_2_)*ρ* to split attractive from repulsive non-covalent interactions.

To determine the position and nature of the interactions, the reduced density gradient isosurfaces are visualized in 3D space. As such, different types of non-covalent interactions, like hydrogen bonds, van der Waals interactions, ionic interactions and repulsive steric clashes, can be analyzed at the same time. Importantly, the NCI index uses the molecular density and its derivatives, thereby removing reliance on user-defined geometrical cutoffs.

Similarly to conventional methods, the NCI index can be applied to large systems, such as the enzyme AhpE, by using pro-molecular densities that are constructed by adding exponential atomic densities^21^. Hence, the NCI index only requires the knowledge of the atomic coordinates. For model arginine···cysteine complexes, we have analyzed how the use of promolecular densities affects the NCI results and, importantly, the NCI results at the self-consistent and promolecular level are quantitatively equivalent (Table S3 and Fig. S5).

**Figure 3:**
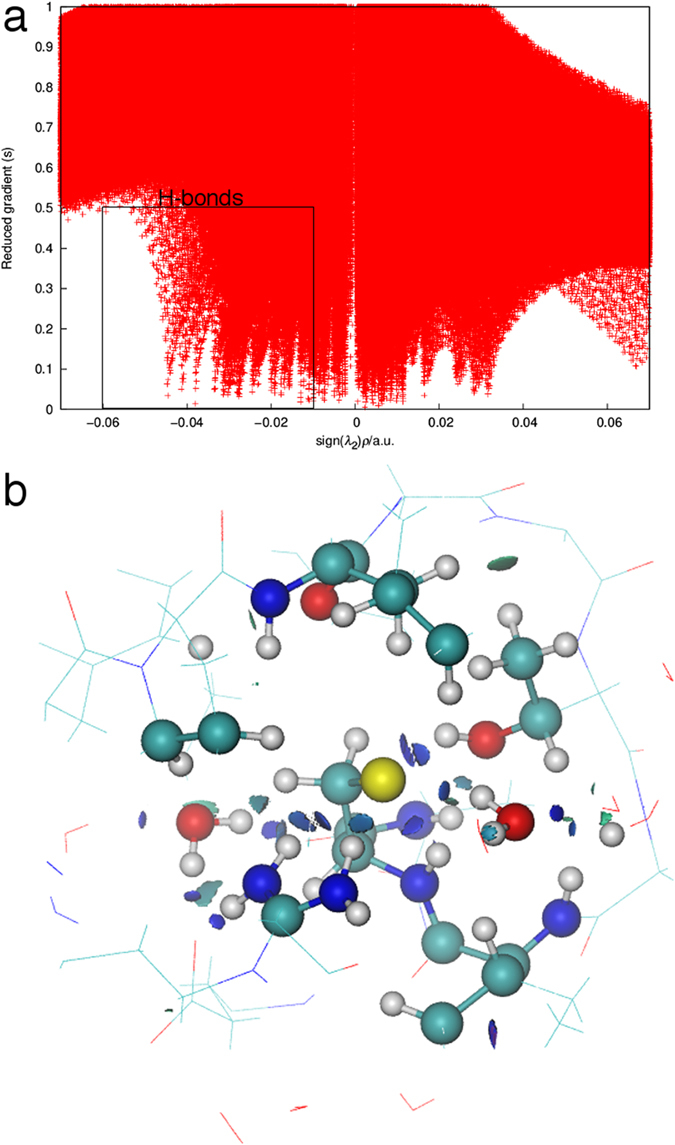
The output from the NCI analysis for the thiolate chain A structure showing the H-bond network. (**a**) The bi-dimensional plot of the reduced density gradient *s* versus sign(*λ*_2_)*ρ* with the H-bond region indicated. (**b**) Gradient isosurfaces (*s* = 0.2 a.u) colored on a RGB scale according to the sign(*λ*_2_)*ρ* values over the range of −0.03 to 0.03 a.u.

**Figure 4:**
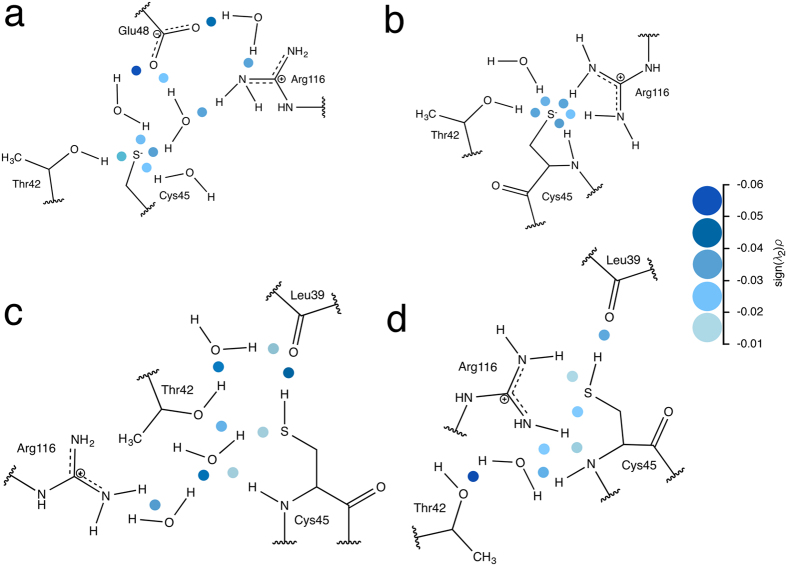
The H-bond networks found in the active site of AhpE by the NCI method. H-bonds are colored in blue and the color intensity is proportional to their strength according to the sign(*λ*_2_)*ρ* values. The strength of the H-bonds ranges from −0.06 to −0.01 a.u. (**a**) Chain A with Cys45 modeled as a thiolate. (**b**) Chain B with Cys45 modeled as a thiolate. (**c**) Chain A with Cys45 modeled as a thiol. (**d**) Chain B with Cys45 modeled as a thiol.

For the active sites of chain A and B of AhpE, we computed *s*(*ρ*) diagrams (example in Fig. 3a) and visualized the corresponding 3D isosurfaces (example in Fig. 3b). Each of these isosurfaces corresponds to one of the low-gradient peaks of the accompanying 2D diagram. By analyzing all the individual peaks with a value of −0.06 a.u. < sign(*λ*_2_)*ρ* < −0.01 a.u., the H-bond network formed by Cys45 was determined (Fig. 4). It immediately became clear that the NCI method finds more sulfur H-bonds than any of the other tested methods. In chain A with Cys45 modeled as a thiolate, the sulfur has four direct H-bonds (Fig. 4a), while in chain B five H-bonds were found (Fig. 4b). As it can be inferred from the sign(*λ*_2_)*ρ* values listed in Table 1, the H-bonds with the sulfur in chain A are slightly weaker in interaction to the ones in chain B, and the strongest interaction in chain B is with the amide of its own peptide backbone (−0.031 a.u.). Due to the presence of an extra H-bond in the active site of chain B, it is likely to be more stable. In the active site of chain B, most H-bonding interactions are intra-protein, involving the side chains of Thr45, Arg116, and the backbone amide of Cys45. In the case of chain A, however, the H-bonding network is much more dependent on water molecules, which, in their mobility, provide a less stable H-bonding environment.

We then analyzed the two chains after simulation with the Cys45 as thiol. In both chains A and B (Fig. 4c,d), Cys45 donates a H-bond to Leu39. This H-bond is relatively strong in chain A (−0.041 a.u.), while it has moderate strength in chain B (−0.028 a.u.) (Table 1). In chain A, the sulfur also accepts a H-bond from a water molecule that bridges Cys45 to Thr42. In chain B, the sulfur accepts two H-bonds from the guanidinium moiety of Arg116.

From this, it can be surmised that the NCI index can distinguish between the H-bonding character of thiol and thiolate in AhpE, with more H-bonds found to the thiolate, than to the thiol. Further, most of the direct H-bonds to Cys45 are stronger for the thiolate (−0.031 a.u. to −0.018 a.u.) than for the thiol (less than −0.02 a.u.).

### An improved equation using an adapted distance criterion

**Figure 5:**
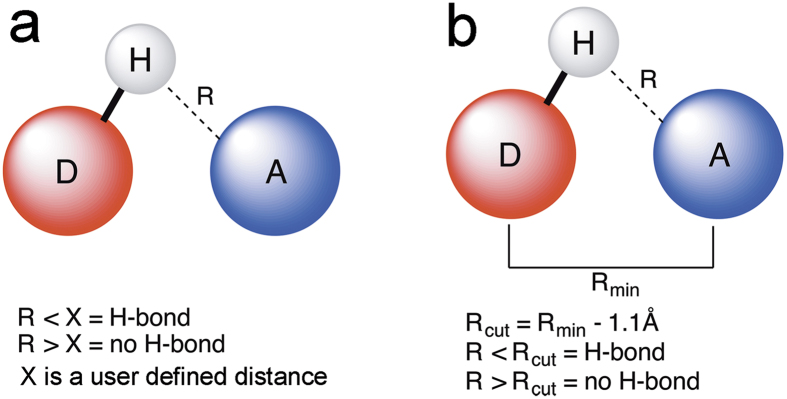
Determining the H-bond network in the active site of the single cysteine peroxiredoxin, AhpE. (**a**) The current established H-bond determination methods^3^,^6^,^7^,^25^ compare the distance between the hydrogen and acceptor atom (R) with a user-defined maximum length. (**b**) The adapted equation uses the minimum van der Waals distance (R_min_) between the donor and acceptor atom to establish the maximum length for a H-bond based on the nature of the atoms. A fixed value of 1.1 Å is subtracted from R_min_. This value was computed by subtracting the distances of the H-bonds found by the NCI index method^21^ from R_min_ for the different pairs of donor and acceptor atoms. The lowest value for R_min_ – R_NCI_ was determined to be 1.1 Å.

By using the NCI method, we showed that the cutoff-distance for H-bonds should be based on the type of heavy atoms involved. We subsequently reasoned that an adapted form of the equation used by conventional methods was needed. To facilitate formulation of such an equation, we employed the program CHARMM, a highly versatile and widely-used molecular simulation program^3^, which we applied here for identifying H-bonds with Method 1. CHARMM uses van der Waals radii for each type of atom A, defined as 1/2*R*_min,A_ (Fig. 5a), where *R*_min,A_ is the minimum of the Lennard-Jones potential between two atoms A. We therefore calculated the distances between the donor hydrogen and the acceptor atom in several H-bond pairs in the re-refined AhpE structures, as found by the NCI index (Table S5), and subtracted this distance from the combined van der Waals radii (*R*_min_) in the program CHARMM (Fig. 5). From this, we found that instead of using a fixed cutoff, one should use *R*_cut_ = *R*_min_−1.1 Å (Fig. 4b), where *R*_min_ = ½(*R*_min,D_ + *R*_min,A_). With this equation almost all H-bonds, which were detected using the NCI method, are also detected using this *R*_cut_ distance criterion. This equation will become particularly useful for analyzing water molecule-mediated H-bonds between several different atom types in the same H-bond network. It is noteworthy that the hydrogen bonding analysis module in the CHARMM program will be updated with this equation in the next version (version 41b).

## Discussion

### Why standard methods are not suited to the characterization of sulfur H-bonds

The number of H-bond interactions found with the NCI index (Table 1), which include all the bridged H-bonds that have no direct interaction with Cys45, show that Method 2 is not only too strict with regard to sulfur, but also produces the lowest number of H-bonds involving only oxygen or nitrogen (Table 1). There are several H-bonds that fall within the 3.0 Å D
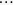
A criteria of Method 2, but the accompanying angle restriction precludes these interactions from H-bond characterization. This highlights the weakness of assigning a more linear nature to H-bonds, as assumed by Method 2. Methods 1 and 3 were more inclusive in their characterization of oxygen and nitrogen H-bonds, but both methods missed a number of H-bonds involving sulfur as acceptor. The H-bonds missed by Method 3 and yet found by Method 1 are attributed to the scaling of the D-H
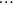
A and H
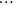
A-X angles.

When comparing the NCI index outcomes with two different set-ups of Method 1 (Fig. S7), it is apparent that a mixed setting of distance cutoffs of the H-bonds, taking the atom types into consideration, gave a more reliable result. When comparing the new distance criterion equation for Method 1 with the NCI index, it is apparent that the overall H-bond networks are very similar. However, the ranking of the individual interactions according to their strength is still completely different (Table S4). Method 1 can only use the distance of H
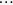
A as a criterion to assess the H-bond strength, while the NCI index uses a density-based strength ranking; the larger the density of the low-gradient spike, the stronger the H-bond. For example, in chain A, Method 1 does not find the water molecule that bridges Cys45 and Glu48, despite this being the strongest interaction found by the NCI index. Conversely, Method 1 finds an extra H-bond between the cysteine sulfur and its backbone amide, which clearly can be ruled out on the basis of the NCI index due to a large steric clash (Fig. S8).

### Comparing the geometrical features of H-bonds found by the NCI method

The H-bonds that are formed with the sulfur as thiol acceptor are, on average, longer (2.74 Å) than the H-bonds with the sulfur as thiolate acceptor (2.34 Å) (Table 1). They are not only longer in distance, but also weaker compared to the sulfur as thiolate. The NCI index revealed six H-bonds were the sulfur was the acceptor, which were not detected by the other methods. From this, it is evident that many H-bonds involving redox-sensitive cysteine residues, with possible catalytic or structural roles, may have been overlooked in the past. In conclusion, the NCI index revealed that sulfur H-bonds can be considered to be at the edge between conventional and unconventional H-bonds, with some H-bonds too weak energetically or too long in interatomic distance to be found with the standard methods.

### Hydrogen peroxide prediction

**Figure 6:**
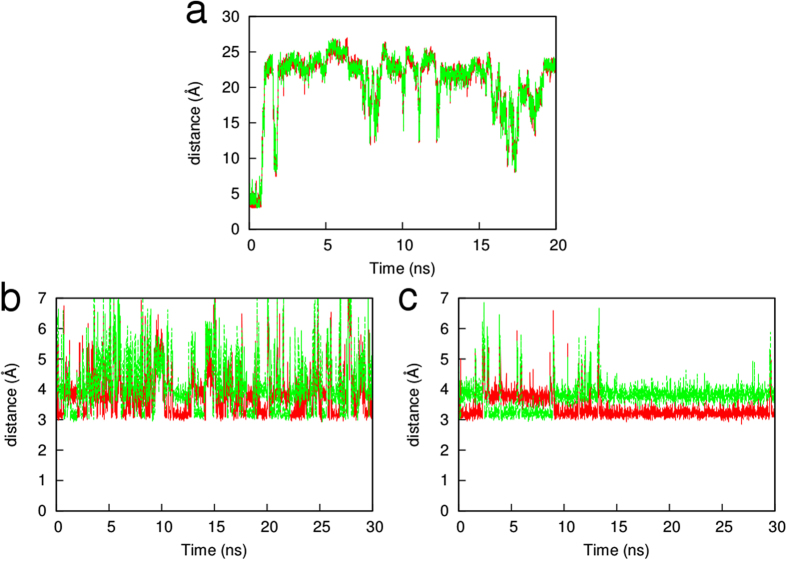
Fluctuations in the distances between the H_2_O_2_ oxygen atoms and the catalytic cysteine S^−^ atom in AhpE: (**a**) unrestrained MD simulation, (**b**) restrained MD simulation on Chain A and (**c**) restrained MD simulation on Chain B.

AhpE reduces H_2_O_2_ to water but no H_2_O_2_ is present in the active site of the AhpE structures. In view of the high similarity between these two molecules, it is difficult to learn about the role of H_2_O_2_ directly by including it in classical MD simulations when a water environment is present. In fact, unrestrained simulations of AhpE with H_2_O_2_ as a ligand in the active site resulted in detachment of H_2_O_2_ from the active site (Fig. 6a). The active site of AhpE is a shallow surface with waters moving quickly in and out during the MD simulation; the unrestrained H_2_O_2_, with force-field parameters very similar to those of water, behaves in a similar manner.

**Figure 7:**
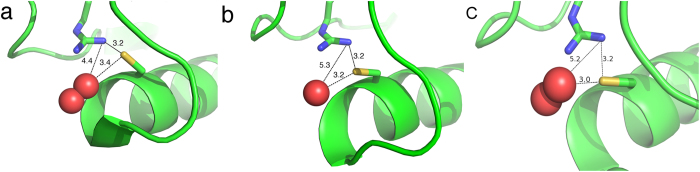
The predicted location of hydrogen peroxide. (**a**) The Prx from *Aeropyrum pernix K1* with H_2_O_2_ crystalized in the structure (PDB: 3a2v)^23^. (**b**) The most likely water in chain B of AhpE predicted to be replaced by H_2_O_2_. (**c**) The location of H_2_O_2_ in AhpE upon a restrained simulation. The H-bond distances (in Å) from H_2_O_2_/H_2_O to the cysteine and arginine are indicated.

However, this similarity between the parameters of H_2_O_2_ and H_2_O can also be used as an advantage. By looking at the positions of waters in the active site during simulation, the most critical docking site of the H_2_O_2_ in the AhpE active site can be identified. In the case of Cys45 as thiol, neither in chain A nor in chain B has a water molecule Cys45 H-bond been found, so it is highly unlikely that the thiol form is the actual form that will react with H_2_O_2_. This is in line with the experimental findings of a pK_a_ of 5.2 for the thiol in the reduced enzyme^22^. In the case of Cys45 as thiolate, both chains have sulfur-water H-bonds. For chain A, the most plausible binding site is the water molecule that is not interacting with any other part of the protein. This water H-bonds to the sulfur with a strength of only −0.018 a.u. For chain B, there is only a single water molecule that H-bonds with sulfur, and it does so with a strength of only −0.024 a.u. These two relatively weak H-bonds are the most likely positions for hydrogen peroxide to displace a water molecule in the reaction mechanism (Fig. 7), which can be compared with the position of H_2_O_2_ bound in the crystal structure of *Aeropyrum pernix K1*^23^ (Fig. 7a).

In order to confirm our prediction, we performed a distance-restrained MD simulation for the H_2_O_2_-AhpE system, in which the distance between an oxygen atom of the ligand and the sulfur of Cys45 was energetically set to be favorable between 3.2 and 3.6 Å. Through this NOE distance restraint, the H_2_O_2_ remained in the active site during the 30 ns simulation.

A plot of the distance between the sulfur and each of the oxygen atoms of H_2_O_2_ (Fig. 6c) along the trajectory shows that the distance remains constant around 3 Å, though only in the case of chain B. For chain A (Fig. 6b), there are large fluctuations in both distances, which is in agreement with findings that chain B is most likely the active form^24^. Importantly, the position of H_2_O_2_ in chain B (Fig. 7c) is very similar to the location of the weakly bonded water molecule predicted by the NCI method (Fig. 7b), as well as crystallized H_2_O_2_ in the structure of Prx from *Aeropyrum pernix K1* (Fig. 7a). This result confirms that the individual strength of the H-bonds provided by the NCI index could be very useful in predicting the position of the ligand in the active site.

### Significance

Most of the established methods to find H-bonds in proteins have user-defined cut-offs, usually specified for classical H-bonds involving oxygen and nitrogen atoms. The moment sulfur is involved; the established methods fail to entirely detect all possible H-bonds. To address such an oversight, we have used the NCI index method. This method is user-independent and probes for the strength of individual H-bonds. To test our approach on structural coordinated atoms in space, we used the active site environment of the peroxidatic sulfur of peroxiredoxin AhpE, a single cysteine enzyme. When using a range of conventional methods, we systematically failed to detect all possible H-bonds of this sulfur, which were eventually detected by applying the NCI method. This observation lead to the development of an improved equation using an adapted distance criterion taking the nature of the atoms into account. In general, this finding will lead to the re-evaluation of all conventional methods in which sulfur H-bonds are potential concern. Independent of this finding, we showed that the NCI index, which is well established for small molecules, is also applicable to proteins. Furthermore, we used the NCI method to determine which water molecule is most likely to be replaced by hydrogen peroxide, thereby predicting the most plausible location of the ligand-binding site in the reduced AhpE. Beyond that, our results confirm that chain B, where the conserved Arg116 is located in the surroundings of Cys45 active site, is the reactive conformation for H_2_O_2_ reduction.

## Methods

### MD setup

For both the original and re-refined structures, chain A was modeled with either thiol or thiolate at Cys45 and hydrogen atoms were added using CHARMM^3^,^25^, version37b1, with the all-atom CHARMM22 force field^26^. The parameterization of the CHARMM force field consists of obtaining appropriate intramolecular, van der Waals and electrostatic parameters that reproduce the selected target data^27^. The corresponding force-field parameters for the thiolate form of Cys were transferred from the methyl-thiolate as a model compound^15^. Importantly, the CHARMM force field was previously used in MD simulations of a glutaredoxin and related proteins to establish the structure, dynamics and electrostatics of active-site cysteine residues^15^. Analysis of the H-bond network combined with experimental p*K*_a_ values revealed an important dependence of the thiolate p*K*_a_ and redox properties on the number of hydrogen bonds involving the thiolate.

All structures were encompassed by a sphere of TIP3P^28^ water molecules with a radius of 35 Å with the sulfur of the cysteine as the center. Since a fixed zone beyond 25 Å was used in the simulations, no counter-ions were added, giving systems with a net charge of either −4 for the thiol, or −5 for the thiolate. The reaction zone was defined as a sphere with a radius of 21 Å from the center^3^. In the shell between the radii of 21 and 25 Å, the atoms were harmonically constrained with an increase in constraints per Angstrom step outwards. Beyond a radius of 25 Å all atomic positions were kept constant. Water molecules were energy-minimized, followed by a short heating step from 10 K to 300 K in 1 ps and an equilibration of 20 ps, and then a second energy-minimization. From this, the whole structure was energy-minimized by 6, short, 500-step cycles of steepest descent minimization and by an adopted basis Newton-Raphson minimization. The systems were heated up gradually to 300 K at the pace of 5 K/ps, followed by 100 ps of equilibration.

The 30 ns simulation run for the opening of the helix was conducted with the Leapfrog Langevin dynamics with a time step of 1 fs, and all X-H bonds were constrained with the SHAKE algorithm^29^. These simulations were performed at constant temperature of 300 K. Every 35 steps the atoms were checked to be in the Langevin region with a 21 Å radius. Energies of the system were written out every 100 steps, coordinates and velocities of the system were written out every 500 steps. A list of non-bonded interactions was updated based on a heuristic testing with a cutoff of 14 Å. Atom electrostatics were treated with a classical force shift method, and the van der Waals interaction forces and energies were set to zero at the cutoff distance using a shift method with 12 Å cutoff.

Unrestrained simulations of 30 ns for the H_2_O_2_-AhpE were performed following the same procedure. For the restrained simulations, a restraining potential was set on the distance between the sulfur of Cys45 and one of the oxygen atoms of H_2_O_2_. The restraint was a half-harmonic potential:


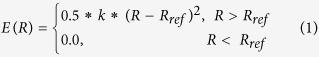


with *k* = 75.0 kcal mol^−1 ^Å^−2^ and *R*_*ref*_ = 3.6 Å. *R*_*ref*_ was based upon the S···O distances involving the peroxidatic sulfur in the *Aeropyrum pernix* K1 structure in its peroxide-bound form^23^. It is noteworthy that similar restrained MD simulations restraining the S
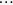
O distance were recently performed to explore the reaction mechanism of peroxiredoxins with hydrogen peroxide^30^.

### Locating H-bonds

We used the equilibrated structure from the MD simulation to observe the H-bond network at the start of the simulation. Every solvated structure of both chain A and B in either thiolate or thiol Cys45 was a single pdb file independent of all other structures. The method using one geometrical constraint was performed by CHARMM^3^ with the standard setting of H
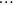
A distance at a maximum of 2.4 Å (Method 1). The method using two geometrical constraints was performed by VMD^6^ using the standard settings of D
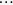
A distance at a maximum of 3.0 Å and D-H
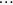
A angle between 160° and 180° (Method 2). The pseudo-energy method was performed by YASARA^7^ at the standard cutoff of 6.25 kcal/mol (Method 3).

### NCI method

NCI plots were computed with the NCIPLOT program using promolecular densities since electron density calculations for the AhpE structures are very expensive computationally^21^. The promolecular densities are computed from the sum of atomic contributions taking the atomic positions into account. In the NCIPLOT program, atomic densities were fit to one (H-He), two (Li-Ne) or three (Na-Ar) Slater-type functions since the use of exponential functions to build the promolecular density allows first and second derivatives to be obtained analytically^11^. In such a way, the reduced density gradient (*s*) together with the second eigenvalue of the electron density Hessian matrix (*λ*_2_) can be very efficiently computed for large systems. For computing the NCI plots, we defined a cube with a side length of 20 Å with the peroxidatic sulfur at the cube center (Fig. S3), in which the promolecular density (*ρ*) and the reduced density gradient (*s*) was computed. The latter is given by:


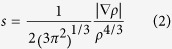


The representation of *s* versus sign(*λ*_2_)*ρ* shows representative peaks at low density values in the presence of non-covalent interactions due to the annihilation of the reduced density gradient at these points^31^. From the density values of the low gradient spikes, the strength of the individual interactions is derived, whereas the second eigenvalue of the electron-density Hessian matrix (*λ*_2_) is used to distinguish between attractive (*λ*_2_ < 0) and repulsive interactions (*λ*_2_ > 0). The sign of the Laplacian (∇^2^ *ρ*) is a widely used tool to distinguish between the different types of strong interactions^32^, since it determines whether the density is concentrated (∇^2^ *ρ* < 0) or depleted (∇^2^ *ρ* > 0) at one point relative to the surroundings. However, for weaker non-covalent interactions, the Laplacian in the interatomic region is dominated by the positive contribution regardless of the whether they are bonding or nonbonding interactions^21^. To distinguish between attractive and repulsive interactions, the NCI approach considers the accumulation or depletion of density in the plane perpendicular to the interaction, which is mainly characterized by the second eigenvalue of the electron-density Hessian matrix. By using the three components along the three principal axis of the maximal variation, the Laplacian can be written as follows:


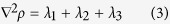


with *λ*_i_ (i = 1, 2, and 3) being the three eigenvalues of the electron density Hessian matrix.

A very important tool is the visualization of the reduced density gradient isosurfaces in real space using VMD^6^. The isosurfaces are colored according to their relative strength using the value of the sign(*λ*_2_)*ρ*. A RGB-color scale (red-green-blue) is normally used, where red isosurfaces represent repulsive interactions, green very weak van der Waals interactions and blue attractive interactions.

## Additional Information

**How to cite this article**: van Bergen, L. A. H. *et al.* Revisiting sulfur H-bonds in proteins: The example of peroxiredoxin AhpE. *Sci. Rep.* **6**, 30369; doi: 10.1038/srep30369 (2016).

## Supplementary Material

Supplementary Information

## Figures and Tables

**Table 1:** Geometrical data for the H-bonds found with the NCI method.

Amino acids		Data				Method		
Donor	Acceptor	H 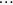 A (Å)	D 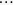 A (Å)	D-H 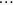 A (°)	Strength (a.u.)	1	2	3
Chain A – thiolate form								
H_2_O	Glu48 Oε	1.72	2.67	174.0	−0.048	x	x	x
H_2_O	Glu48 Oε	1.88	2.77	152.6	−0.037	x		
Arg116 Hη	H_2_O	2.01	2.90	146.7	−0.028	x		x
H_2_O	Glu48 Oε	2.13	2.99	149.3	−0.022	x		x
H_2_O	Arg116 Nη	2.15	3.10	169.3	−0.027			
H_2_O	Cys45 Sγ	2.20	3.15	175.5	−0.030	x		x
Thr42 Hγ	Cys45 Sγ	2.29	3.18	153.2	−0.026	x		x
H_2_O	Cys45 Sγ	2.44	3.34	157.3	−0.022			
H_2_O	Cys45 Sγ	2.49	3.45	176.6	−0.018			
Chain B – thiolate form								
Cys45 H	Cys45 Sγ	2.27	3.00	128.5	−0.031	x		
Arg116 Hη	Cys 45 Sγ	2.30	3.22	151.3	−0.028	x		
H_2_O	Cys45 Sγ	2.31	3.21	157.5	−0.024	x		x
Thr45 Hγ	Cys45 Sγ	2.33	3.18	146.5	−0.028	x		
Arg116 Hη	Cys45 Sγ	2.45	3.33	146.8	−0.020			
Chain A – thiol form								
Cys45 Hγ	Leu39 O	1.80	3.03	152.1	−0.041	x		x
H_2_O	H_2_O	1.84	2.74	156.4	−0.037	x		x
Thr42 Hγ	H_2_O	1.90	2.84	168.1	−0.035	x	x	x
Arg116 Hη	H_2_O	1.98	2.92	155.7	−0.031	x		x
H_2_O	Thr42 Oγ	2.02	2.93	157.1	−0.026	x		x
H_2_O	Leu39 O	2.35	3.27	162.4	−0.016	x		
Cys45 H	H_2_O	2.51	3.33	138.8	−0.012			
H_2_O	Cys45 Sγ	2.77	3.22	109.4	−0.012			
Chain B – thiol form								
H_2_O	Thr42 Oγ	1.71	2.65	164.0	−0.052	x	x	x
Cys45 Hγ	Leu39 O	2.00	3.10	136.6	−0.028	x		x
Cys45 H	H_2_O	2.05	2.93	146.1	−0.026	x		x
Arg116 Hη	H_2_O	2.24	2.95	126.9	−0.018	x		x
Arg116 Hη	Cys45 Sγ	2.49	3.31	138.8	−0.018			
Arg116 Hη	Cys45 N	2.57	3.33	132.3	−0.015			
Arg116 Hη	Cys45 Sγ	2.95	3.65	130.9	−0.010			

The distances H
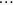
A and D
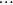
A are in Å. The D-H
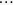
A angle is in degrees (°). The strength [sign(λ_2_)*ρ*] of the individual H-bonds provided by the NCI method are given in a.u. In the last three columns of the table, it is indicated whether the three conventional methods were able to find these H-bonds.
