# Supplementary material for: Revisiting sulfur H-bonds in proteins: The example of peroxiredoxin AhpE
Source: Sci Rep. 2016 Jul 29;6:30369. doi: 10.1038/srep30369 (PMC4965862; doi:10.1038/srep30369)
Supplement: Supplementary Information [file srep30369-s1.pdf]

Supplementary information

Revisiting sulfur H-bonds in proteins: The example of peroxiredoxin AhpE

Authors:

Laura A. H. van Bergen [1,2,3,4], Mercedes Alonso[1], Anna Palló[2,3,4], Lennart Nilsson[5], Frank De Proft[1]*, and Joris Messens [2,3,4]*

Affiliations:

[1]Research Group of General Chemistry, Vrije Universiteit Brussel, 1050 Brussels, Belgium

[2]Structural Biology Research Center, VIB, 1050 Brussels, Belgium

[3]Brussels Center for Redox Biology, 1050 Brussels, Belgium

[4]Structural Biology Brussels, Vrije Universiteit Brussel, 1050 Brussels, Belgium

[5]Department of Biosciences and Nutrition, Karolinska Institutet, SE-14183 Huddinge, Sweden

Contact:

Joris Messens:

Structural Biology Research Center, VIB - Vrije Universiteit Brussel
Pleinlaan 2, B-1050 Brussels, Belgium
Tel.: +32 2 6291992; Fax: +32 2 6291963; E-mail: joris.messens@vib-vub.be

Frank De Proft:

Research Group of General Chemistry (ALGC), Vrije Universiteit Brussel (VUB)
Pleinlaan 2, B-1050 Brussels, Belgium
Tel.: +32 2 6293310; Fax: +32 2 6293317; E-mail: fdeprof@vub.ac.be

Supplementary results

Re-refinement of the models for AhpE improves protein stability during MD simulations

To study the interactions of the sulfur as a hydrogen bond donor or acceptor atom, we used the enzyme Alkyl hydroperoxide reductase E (AhpE) from *Mycobacterium tuberculosis* as a case study, since it is a one-cysteine (Cys45) peroxiredoxin (Prx) and the protein is relatively moderate in size (153 amino acids). AhpE is part of the defense mechanism of this pathogen through its ability to detoxify peroxides through sulfur oxygen chemistry on its cysteine. The accompanying reaction mechanism has been well characterized, thus allowing the validation of our computational results. The active site of AhpE is of particular interest, since it is one of the few in its family that has no resolving cysteine. Furthermore, the Cys pK_a (5.2) suggests that the majority of the cysteine population should be present in the thiolate form[1].

Both the reduced (PDB: 1xxu) and the sulfenylated (PDB: 1xvw) form have been crystalized[2]. AhpE is a monomer in solution, but the crystal structure shows 2 dimers per asymmetric unit, which are denoted chains A and B. We decided to use both of these chains in our analysis, because the cysteine environment in both chains is different. In chain A, the side chain of the conserved arginine residue (Arg116) is located away from the cysteine and directed outwards to the flexible β9-α5 loop, while in chain B it is turned inwards to the active site cysteine (Fig. S1). For other peroxiredoxins, the inwards orientation, as observed in chain B, has been described as the active form, and the activity of the enzyme has been coupled to the movement of Arg116[3,4]. However, after inspection of the electron density maps of the structures available in the PDB, we observed that there are several questionable sites, justifying a re-refinement of the protein structures.

We started by looking at the updated AhpE structures found in the PDB REDO database[5], since they were found to be significantly better than the ones in the PDB. After several cycles of manual re-modelling and REFMAC[6]

re-refinement, the new structures of the reduced and sulfenylated AhpE were deposited to the PDB under accession codes 4x0x and 4x1u, respectively.

The R-factors decreased with 3%, so our new models of AhpE are an improved representation of the experimental data (Table S1). Based on the electron density maps and taking into account the chemical environment, ten highly improbable amino acid rotamers per chain were optimized, and several side chains were flipped (Table S2). Five molecules of the cryo-protectant glycerol and several missing water molecules (158 in case of 1xxu, and 54 in case of 1xvw) were added, and they all contributed to the improvement of the model and the decrease of the R-factors. To check whether the fitting of the protein chains to the density map also would improve the R-factor, we performed another REFMAC refinement on the re-refined structure in the absence of the small molecules. It turned out that the new waters only account for ~ 0.5 % decrease in the R-factors. So the majority of the improvement is due to the re-refined protein chains.

Important changes include Gln46, the amino acid adjacent to the peroxidatic cysteine. Flipping the side chain of Gln46 allows it to be involved in H-bonding interactions with the side chains of the conserved Trp80, Ser84 and Asp50, which may contribute to the stability of the active site (Fig. S2c). The new interaction to Trp80 is of special importance, since this residue is supposed to be predominantly causing the decrease of fluorescence signal upon sulfenylation of AhpE[1], which effect is used to characterize the kinetic behaviour of the enzyme[7]. According to the crystal structures, the main change in the environment of Trp80 upon oxidation of AhpE is the change in oxidation state of Cys45. As the Cys45 side chain lays only 5 Å away from the benzene part of the indole ring, it can influence the electron density of this Trp80.

Gauche minus is the active conformation of the peroxidatic cysteine

In the active site of chain A of the re-refined reduced AhpE structure(4x0x), the electron density map implies a different conformation of the peroxidatic Cys from the one in the PDB. In 1xxu, Cys45^{A} was modeled in gauche plus

conformation[2], with χ_1 of +61°; however, both the $2F_o$-F_c and the F_o-F_c density maps showed that the gauche minus conformation is more probable (Fig. S2a). At an rmsd level of 3, a small negative peak on the F_o-F_c difference density map was observed at the original position of the Cys45 side chain, while a positive peak on the map suggested a possible alternative location of the side chain. Therefore, Cys45^A was modeled both as adopting only the gauche minus conformation, and as a 50-50% mixture of the gauche minus and plus conformers, and both structures were subjected to 10 REFMAC refinement cycles. The refined density map supports the gauche minus form as the most probable one.

As an alternative, Cys45 was also modeled and refined in its oxidized form as a sulfenic acid, but this results in a negative peak in the difference density map. Apart from that a positive peak in the difference density map was observed 2.6 Å away from the side chain of Cys45^A (Fig. S2a), similarly to its non-crystallographic symmetry mate, the Cys45^D. Therefore, these densities were both modeled as water molecules. After this modification, the side chain of the peroxidatic cysteine pointed towards the surface of the protein with the χ_1 torsion angle close to -60°, similarly to the three other protein chains in the asymmetric unit. Based on the analysis of about 80 homologue peroxiredoxin structures of the PDB, it can be concluded that the gauche minus conformation of the peroxidatic cysteine dominates. This conformation was detected in more than 80 % of the peroxiredoxins, including peroxiredoxins in complex with hydrogen peroxide[8], which suggests the gauche minus conformation as being the active conformation of the peroxidatic cysteine.

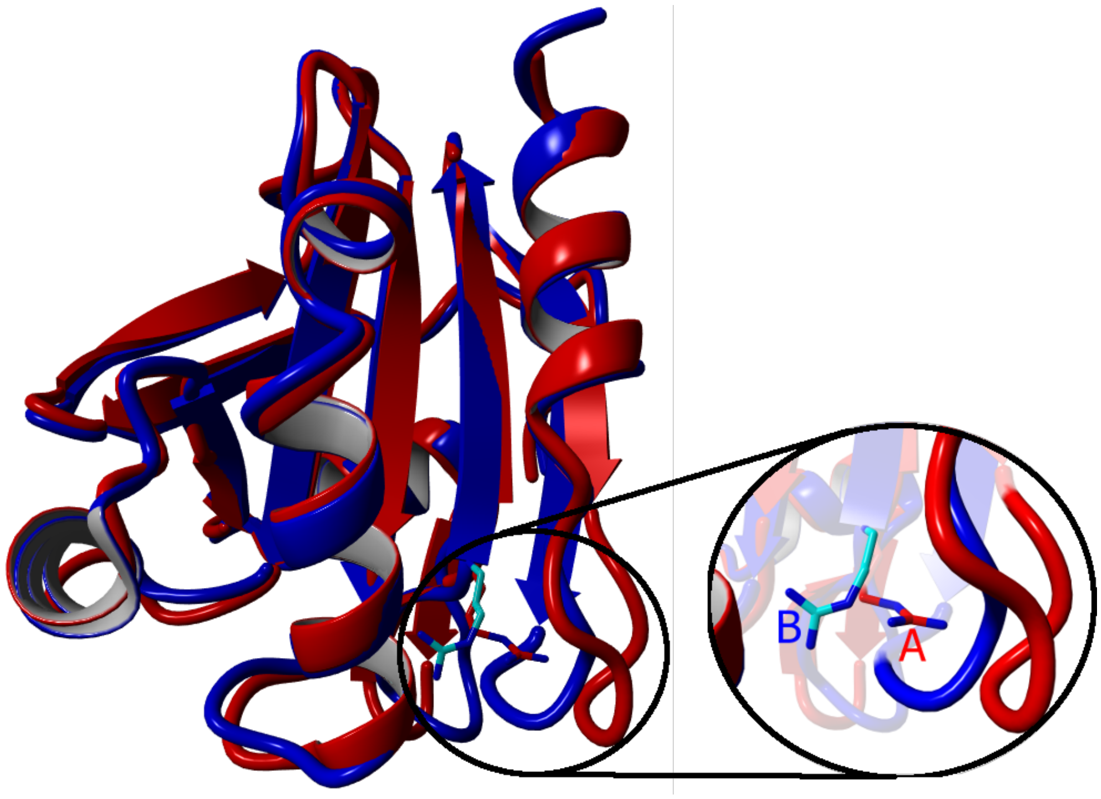

Figure S1. Structural overlay of chains A and B of AhpE. The inset shows the conformational differences between the side chains of Arg116 in the two chains.

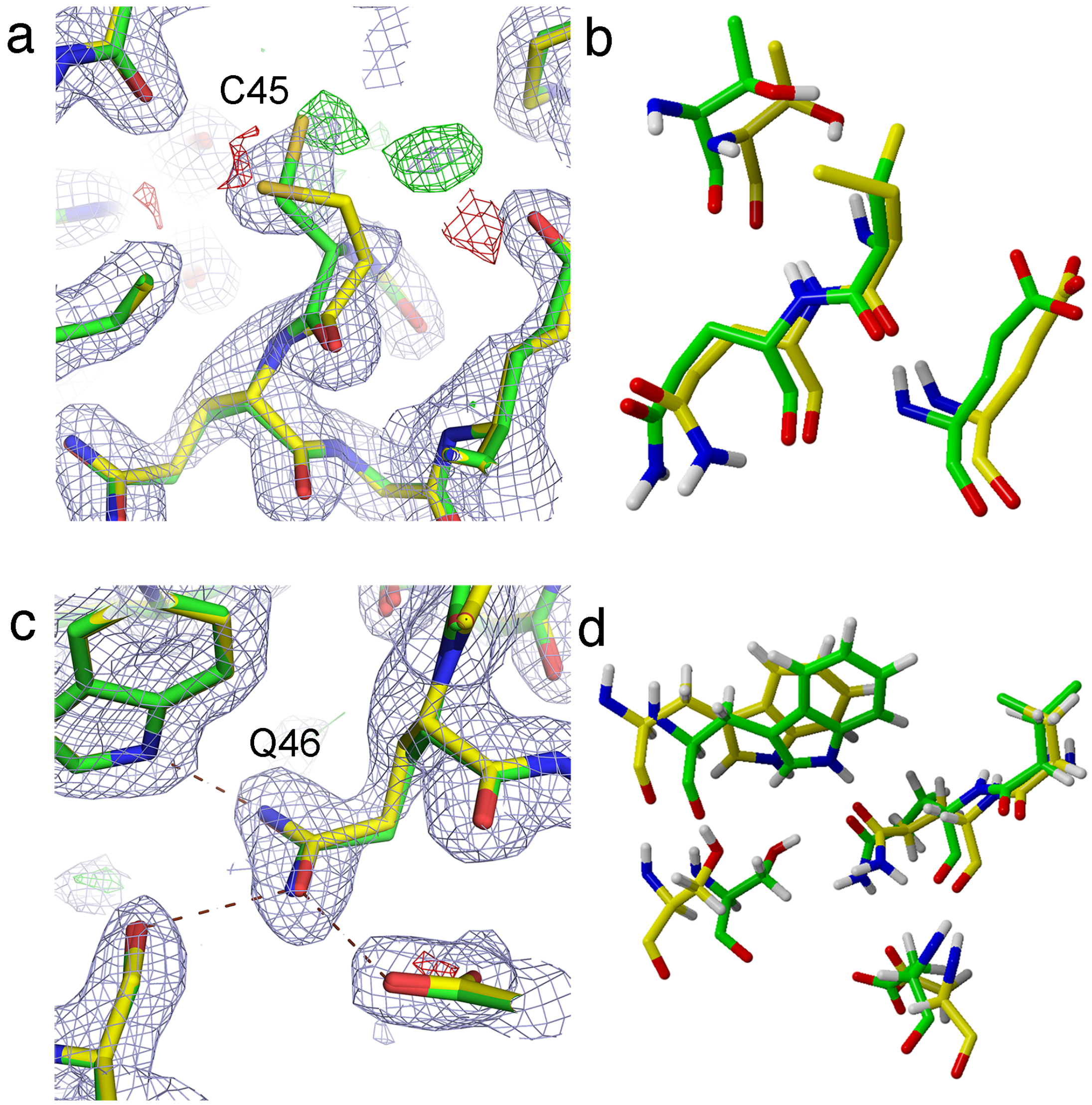

Figure S2 .The conformation of the AhpE active site Cys45 and Gln46 residues after re-refinement and after MD equilibration. The original PDB entry is shown in yellow carbon atoms, while the re-refined structure is shown in green carbon atoms. (**a**) The original structure represents the peroxidatic Cys45 of chain A in gauche plus conformation, while the electron-density maps imply a gauche minus conformation with Cys45 pointing outwards the binding cleft. (**b**) The differences in the conformation of Cys45 from the input structures are retained in the MD equilibrium structures. (**c**) The fitting of Gln46 in the density map at 1.9 Å resolution does not improve by flipping the side chain of Gln46, although it provides a chemically more reasonable interactions with Trp80, Ser84 and Asp50. (**d**) During the MD simulation the active site remains more stable in the re-refined structure than in the original one.

	1xxu	4x0x	1xvw	4x1u
Starting atom	1xxu.pdb	1xxu.pdb	1xvw.pdb	1xvw.pdb
coordinates	1xxu-sf.cif	1xxu-sf.cif	1xvw-sf.cif	1xvw-sf.cif
Structure factors	0.242	0.219	0.180	0.151
$R_{work.}$	0.286	0.249	0.210	0.183
R_{free}				
Number of atoms	4760	4760	2478	2469
protein	0	0	0	5
other organic	187	349	185	239
water molecules				
R.m.s. deviations	0.012	0.018	0.013	0.019
bonds (Å)	1.6	1.7	1.6	1.9
angles (°)				
Average B-factors (Å^2)	21.5	19.4	22.7	24.8
protein	-	-	-	47.1
other organic	28.0	31.4	31.6	35.8
water molecules				
Ramachandran plot	96.4	97.1	96.8	97.5
most favored (%)	0.5	0.5	0.6	0.6
outliers (%)	2.8	0.02	1.6	0.0
Rotamer outliers (%)				

Table S1. Juxtaposition of refinement statistics of the PDB entries 1xxu and 1xvw and our re-refined models (4x0x and 4x1u).

Residue	Chains		Change
	reduced	oxidized	
Asn17	A, B, C	A, B	flip
Gln18	B, C, D	B	new rotamer
Gln19		A	flip
Arg27	A, C, D		new rotamer
Lys30	A, B, D	B	new rotamer
Cys45	A		new rotamer
Gln46	A, B	A, B	flip
Gln51	A, C	A, B	flip
His55	A, B, C	A, B	flip
His97	A, B, C, D	A, B	flip
Ser101	B		new rotamer
Gln102	B	B	flip
Asn108	A, B, C, D	B	flip
Met132	A, D		new rotamer
Val138	B		new rotamer
Gln134		B	new rotamer
Gln141		B	new rotamer
Leu143	A, D	A	new rotamer
Thr152	A	A, B	new rotamer

Table S2. Modifications in the conformation of amino acid residues introduced during the re-refinement of AhpE structures.

Supplementary methods

Re-refinement method

Coordinates and structure factor amplitudes were retrieved from the PDB server. The mmcif file containing structure factors was converted to mtz format using the mmcif2mtz tool using the CCP4 suite[9]. Electron density maps were generated with the help of the Electron Density Server hosted at Uppsala University (http://eds.bmc.uu.se/eds). Maps were visualized by the program COOT[10]. First corrections on the coordinates were done using the server PDB REDO. Each alteration done by PDB REDO[5] was visually inspected and evaluated, and COOT was used for model building. Further refinement runs were performed using REFMAC[6] with the settings recommended by PDB REDO. The qualities of the new models were assessed by MolProbity[11]. The coordinate files of the re-refined reduced and sulfenylated AhpE were deposited to the PDB under accession codes of 4x0x and 4x1u, respectively. Statistics of the refinements are reported in Table S1.

Energy equation for the conventional method

$$E_{HBo} = 25 * \frac{2.6 - Dis_{H-A}}{0.5} * Scale_{D-H-A} * Scale_{H-A-X} \quad (1)$$
$$with\ Scale\ set\ between\ 0\ and\ 1$$

The energy calculated for the H-bond formation (E_{HBo}) depends on the H···A distance with a maximum of 2.6 Å. This number is scaled (multiplied with a number between 0 and 1) depending on the value of the two angles that are connecting via the specific H-bond, D-H···A and H···A-X. $Scale_{D-H-A}$ is set to 0 when D-H···A below 100° and to 1 for 165° until 180°. All values of the angle between 100° and 165° are scaled from 0 to 1. The other angle, H···A-X, sets everything below 85° to a scale of 0, while for angles from 95° to 180° it is set to 1 ($Scale_{H-A-X}$). Values between 85° and 95° are scaled from 0 to 1. When X is a hydrogen atom, the scales are adjusted to a 10° lower range. The cutoff

for E_{HBo} to count a H-bond is 6.25 kJ/mol, implying that the calculated energy needs to be at least 25% of the optimum value for a H-bond.

The Non-Covalent Index (NCI) approach

The NCI approach allows identification of interactions in real space, based on the peaks that appear when plotting the reduced density gradient (s) versus density (ρ). The reduced density gradient is given by

$$s = \frac{1}{2(3\pi^2)^{1/3}} \frac{|\nabla\rho|}{\rho^{4/3}} \qquad (2)$$

Plots of s versus ρ for the points in space in the area of interest will exhibit characteristic low-density low-gradient peaks assigned to the presence of non-covalent interactions. Promolecular densities were used for the NCI calculations since since electron density calculations for the AhpE structures are very expensive computationally. The promolecular densities are computed from the sum of atomic contributions taking the atomic positions into account. In the NCIPLOT program, atomic densities were fit to one (H-He), two (Li-Ne) or three (Na-Ar) Slater-type functions of the form $\rho^{at} = \sum_j c_j\, e^{(-\frac{r}{\zeta_j})}$, since the use of exponential functions to build the promolecular density allows first and second derivatives to be obtained analytically[12]. To obtain the NCI plots, a cubic grid of side length 20 Å with the peroxydatic sulfur at the cube center (Fig. S3) in which the promolecular density (ρ) and the reduced density gradient (s) was computed.

The strength of these interactions is derived from the density value at these low-gradient peaks. When more density accumulates between two different atoms, the stronger the interaction will be, which results in an increase of the density value of the low-gradient spike. Consequently, dispersion interactions, like van der Waals, appear at very low-density values ($\rho < 0.01$ a.u.), whereas stronger interactions, like H-bonds and steric hindrance, appear at higher density values ($\rho > 0.01$ a.u.). In this way, the nature of the atoms involved is taken into account, while in most of the conventional methods the distance of

the H-bond is indicative of its strength: shorter H-bonds means stronger interaction. To distinguish between attractive and repulsive interactions, the NCI method uses the sign of the second eigenvalue of the electron-density Hessian matrix (λ_2): $\lambda_2 < 0$ means bonding interactions, while $\lambda_2 > 0$ defines non-bonding ones. λ_2 is derived from the Laplacian ($\nabla^2\rho$), a widely used tool to distinguish between the different types of strong interactions[13], when expressed as the three components along the three principal axes of an interaction (equation 3).

$$\nabla^2\rho = \lambda_1 + \lambda_2 + \lambda_3 \quad (3)$$

with λ_i (i=1, 2, and 3) being the three eigenvalues of the electron-density Hessian matrix.

The combination of the regions of low density and low reduced gradient (equation 2) with the sign of λ_2 (equation 3) gives a unique pattern for the different type of non-covalent interactions. Therefore, the reduced gradient density (*s*) is plotted against the product of the sign(λ_2) and the electron density function (ρ) to generate a bi-dimensional NCI plot (Fig. S3a). This plot can be divided into three regions for different types of NCI; H-bonds are found in the region of -0.06 a.u. < sign(λ_2)ρ < -0.01 a.u., van der Waals are in the region of -0.01 a.u. < sign(λ_2)ρ < 0.01 a.u. and steric hindrance is found in the region of 0.01 a.u. < sign(λ_2)ρ < 0.06 a.u.

Finally, the low-gradient iso-surfaces are plotted in real space (3D) and the isosurfaces are colored according to their relative strength using the value of sign(λ_2)ρ. A RGB-color scale (red-green-blue) is normally used, where red isosurfaces represent repulsive interactions, green very weak van der Waals interactions and blue attractive interactions (Fig. S3b).

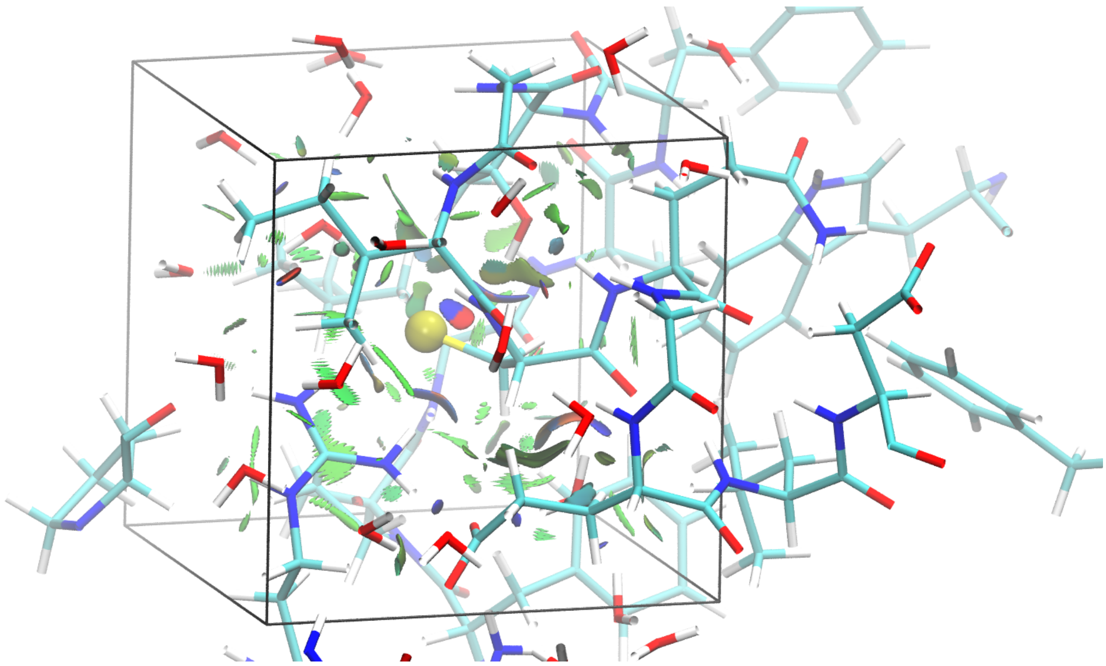

Figure S3 .The NCI calculations were performed within a cube of side length of 20 Å with the peroxydatic sulphur at the center. All the reduced density gradient isosurfaces with a sign(λ_2)ρ value lower than -0.015 a.u. were visually examined to assess if they belong to the H-bond network of the peroxydatic sulfur.

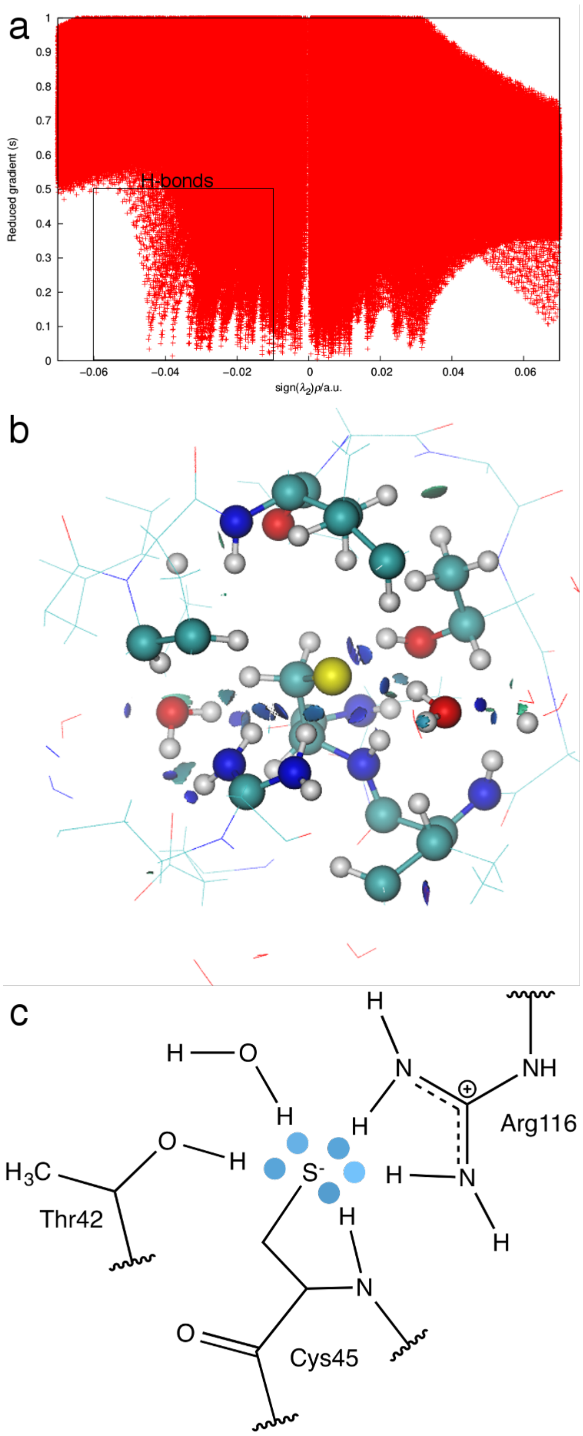

Figure S4. **NCI analysis of the thiolate chain A structure showing the H-bond network.** (**a**) The bidimensional plot of the reduced density gradient *s* *versus* sign(λ_2)ρ with the H-bond region indicated by a rectangle. (**b**) Gradient isosurfaces (s = 0.2 a.u) colored on a RGB scale according to the sign(λ_2)ρ values over the range of -0.03 to 0.03 a.u. (**c**) From the NCI analysis, the hydrogen bonds involving the Cys45 were categorized and represented in 2D maps, where the blue intensity is proportional to the density values of the corresponding NCI peaks.

Computations using the Promolecular Density

In order to assess how the use of promolecular densities affects the NCI results, we have performed NCI calculations for model arginine···cysteine complexes with the peroxidatic Cys as thiolate and thiol forms. The starting geometries for Arg···CysS$^-$ and Arg···CysSH were taken from the equilibrated structure of chain B of the re-refined AhPE. The geometries of both complexes were fully optimized and characterized at the M06-2X/6-311+G** level of theory.[14] All the optimizations were performed with the universal solvation model[15] using a dielectric constant of 20. The use of an implicit solvation model is the most common technique of mimicking the environment at the proteins surface.[16] Then, the NCI calculations were carried out using the self-consistent DFT and the promolecular densities and the results are shown in Figure S5. For both complexes, the NCI results at the self-consistent and promolecular level are quantitatively equivalent. The low-gradient peaks associated to H-bonds remain at very similar density values, as can be inferred from the strength collected in Table S3. The largest shift towards smaller density values were observed for the peaks corresponding to nonbonding interactions upon density relaxation, introducing less repulsion and greater stability. Therefore, the use of promolecular densities becomes a reliable approach to establish the H-bond network of the large AhpE.

	Strength (sign(λ_2)ρ) / a.u.			H-bond distance / Å	
	M06-2X/6-311+G**	Promolecular	Eq. structure	M06-2X/6-311+G**	Eq. structure
CysS$^-$···Arg	-0.029	-0.027	-0.028	2.27	2.30
CysS$^-$···Arg	-0.023	-0.023	-0.020	2.37	2.45
CysSH···Arg	-0.017	-0.016	-0.018	2.50	2.49

Table S3. Influence of promolecular and self-consistent densities on the strength of the H-bonds for cysteine···arginine model complexes with the peroxidatic Cys as thiolate and thiol forms.

In all the cases, stronger H-bonds between the Cys and Arg are found for the thiolate, which is in agreement with the largest interaction energy of the CysS$^-$···Arg complex. The interaction energy computed at the M06-2X/6-311+G** level of theory is reduced from -13.9 kcal mol^{-1} to -2.2 kcal mol^{-1} when going

from the thiolate to thiol cysteine. In addition, the N-H···S$^-$ H-bonds involving Cys45 and Arg116 have lifetimes of 95.6% and 99.8% along the 30 ns trajectory obtained for the MD simulation of AhpE Chain B.

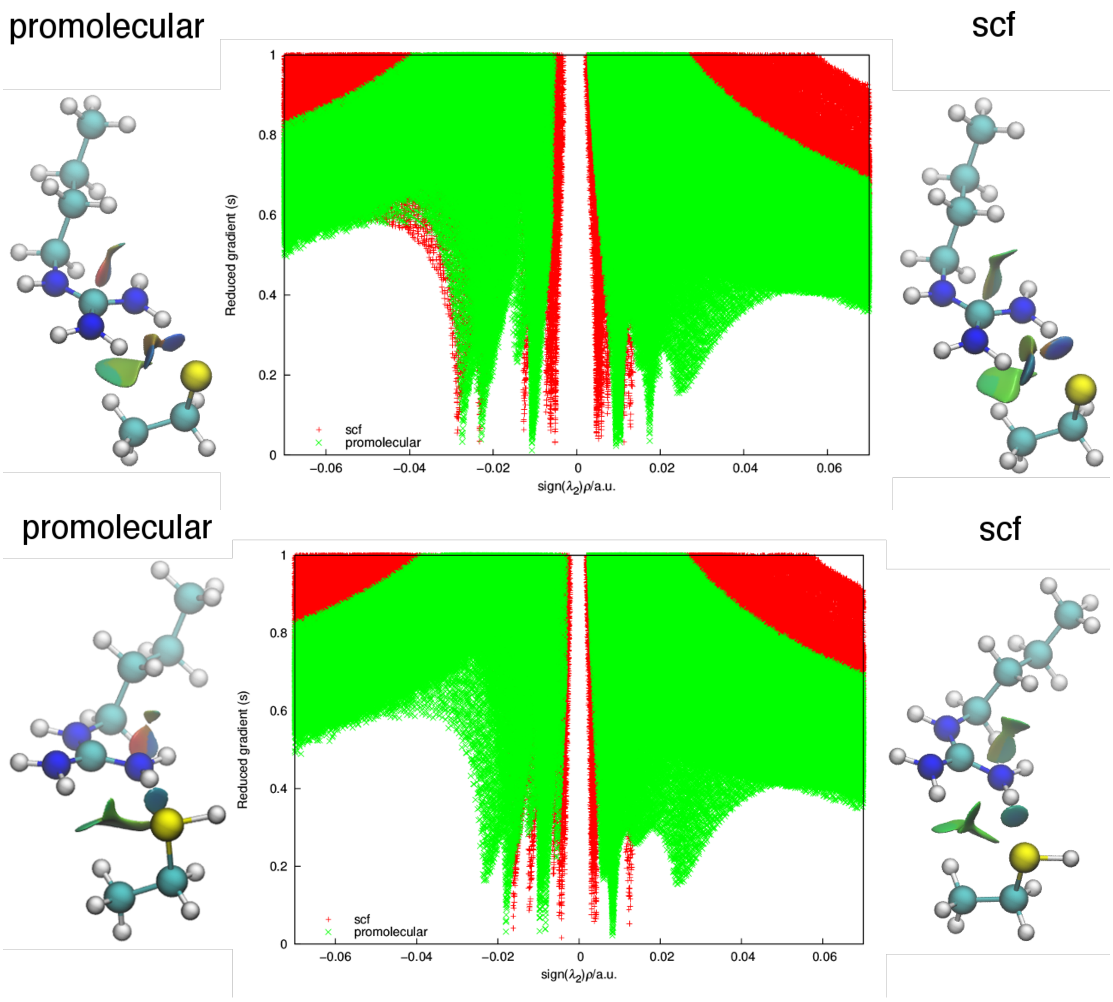

Figure S5. **The *s(ρ)* features of the cysteine···arginine model complexes with the peroxidatic Cys as thiolate and thiol forms obtained using self-consistent (*s* = 0.5) and promolecular densities (*s* = 0.3).** The gradient isosurfaces (*s* = 0.2 a.u) are colored on a RGB scale according to the sign(λ_2)ρ values over the range of -0.03 to 0.03 a.u.

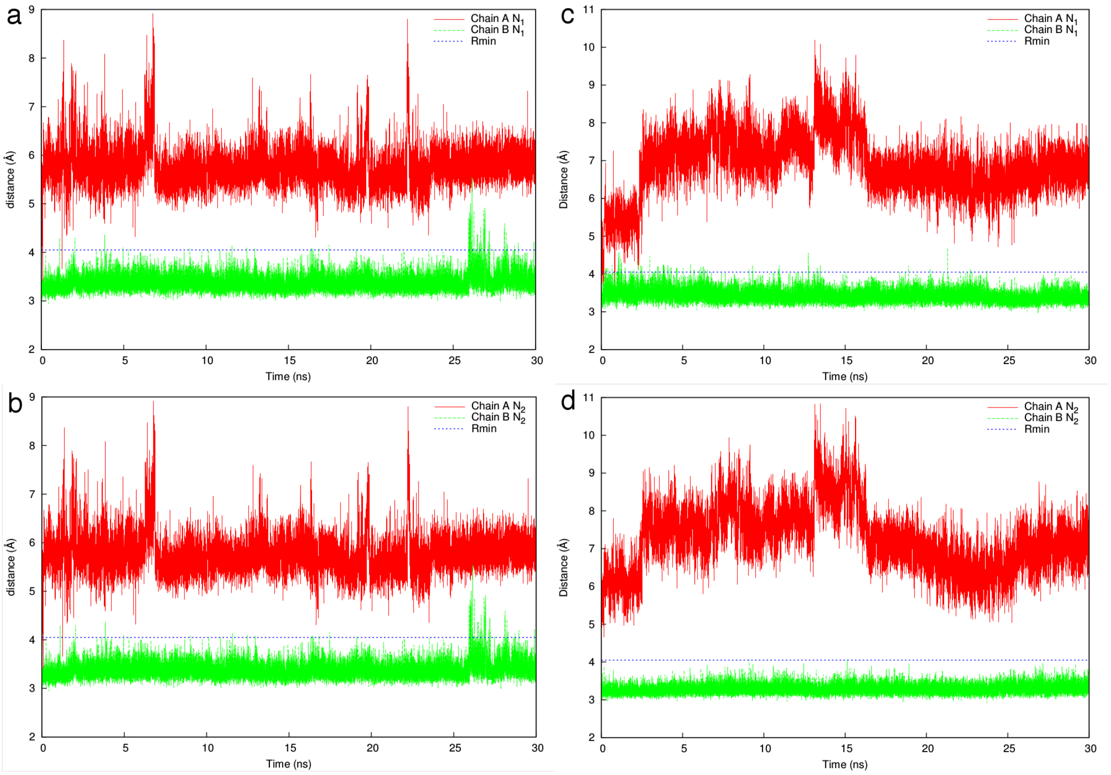

Figure S6. Time-course of H-bond distances between the peroxidatic sulfur in thiolate Cys45 and the nitrogen atoms of the guanidinium moiety of the conserved Arg116 for both chain A (red) and B (green). The R_{min} to establish the cutoff for our new developed equation is also shown (blue), having a value of 4.05 Å for S-···N bonds. Each panel is a comparison of a single H-bond in the two different chains. The left panels are measured over the AhpE trajectories without the H_2O_2 ligand (a, b) and the right panels correspond to the trajectories for AhpE-H_2O_2 constrained simulations(c, d).

Supplementary Figures and Tables

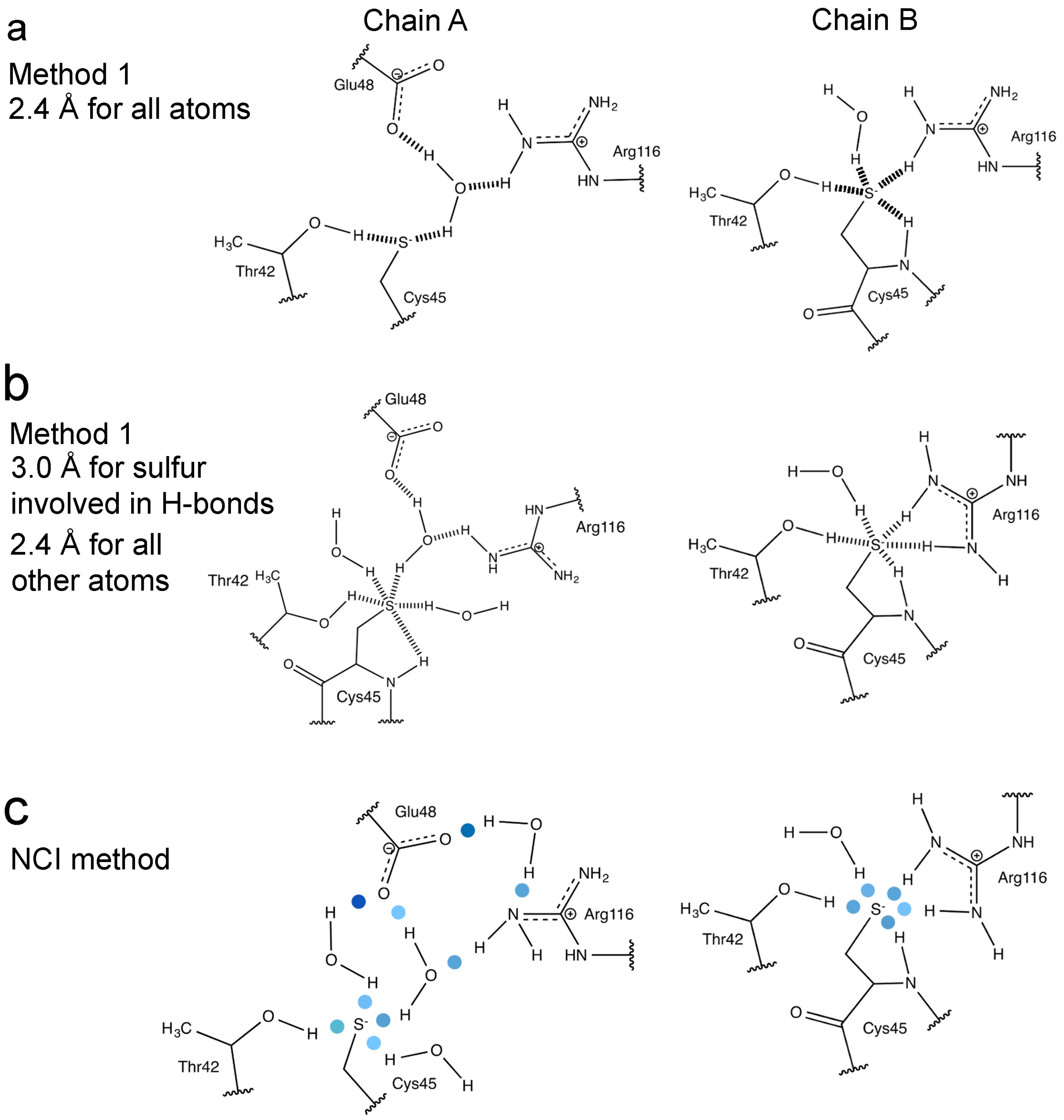

Figure S7. Comparison of H-bond networks in the thiolate chains with different methods. (**a**) The H-bonds network found by Method 1 with all cutoffs set at 2.4 Å. (**b**) Method 1 with cutoffs for sulfur-centered H-bonds of 3.0 Å and the rest set at 2.4 Å, and (**c**) the H-bonds determined with the NCI method**.** The strength of the individual H-bonds is collected in **Table S4.**

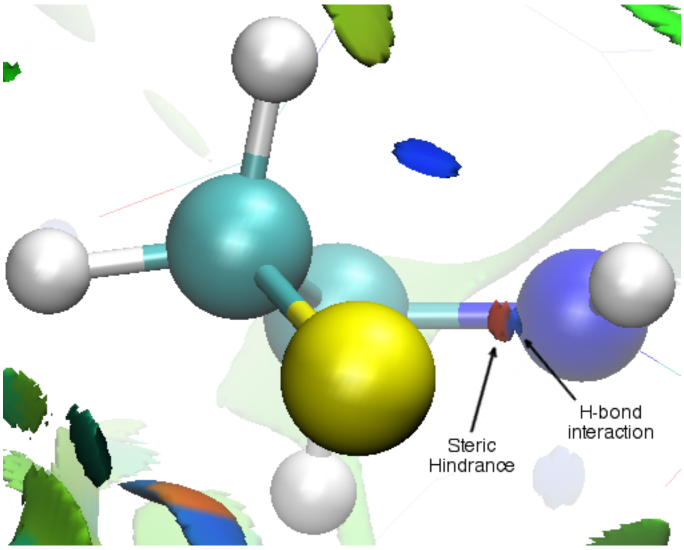

Figure S8. Steric hindrance between the sulphur atom and its NH backbone, revealed by the NCI method. This interaction is regarded as a H-bond by conventional methods. However, the weak H-bond interaction is negated by the large steric hindrance.

Method 1 – 2.4 Å for all atoms			Method 1 – 3.0 Å for sulfur			NCI method		
Donor	Acceptor	Distance	Donor	Acceptor	Distance	Donor	Acceptor	10^2sign(l_2)r
				Chain A				
Arg116 Hη	H_2O (1)	2.00	Arg116 Hη	H_2O (1)	2.00	H_2O (2)	Glu48 Oε	-4.8
H_2O (1)	Glu48 Oε	2.12	H_2O (1)	Glu48 Oε	2.12	H_2O (4)	Glu48 Oε (2)	-3.7
H_2O (1)	Cys45 Sγ	2.20	H_2O (1)	Cys45 Sγ	2.20	H_2O (1)	Cys45 Sγ	-3.0
Thr42 Hγ	Cys45 Sγ	2.29	Thr42 Hγ	Cys45 Sγ	2.29	Arg116 Hη	H_2O	-2.8
			H_2O (2)	Cys45 Sγ	2.44	H_2O (4)	Arg116 Nη	-2.7
			Cys45 H	Cys45 Sγ	2.48	Thr42 Hγ	Cys45 Sγ	-2.6
			H_2O (3)	Cys45 Sγ	2.49	H_2O (1)	Glu48 Oε	-2.2
						H_2O (2)	Cys45 Sγ	-2.2
						H_2O (3)	Cys45 Sγ	-1.8
				Chain B				
Cys45 H	Cys45 Sγ	2.27	Cys45 H	Cys45 Sγ	2.27	Cys45 H	Cys45 Sγ	-3.1
Arg116 Hη (1)	Cys45 Sγ	2.30	Arg116 Hη (1)	Cys45 Sγ	2.30	Thr42 Hγ	Cys45 Sγ	-2.8
H_2O	Cys45 Sγ	2.31	H_2O	Cys45 Sγ	2.31	Arg116 Hη (1)	Cys45 Sγ	-2.8
Thr42 Hγ	Cys45 Sγ	2.33	Thr42 Hγ	Cys45 Sγ	2.33	H_2O	Cys45 Sγ	-2.4
			Arg116 Hη (2)	Cys45 Sγ	2.45	Arg116 Hη (2)	Cys45 Sγ	-2.0

Table S4. The ranking of the H-bonds of the thiolate structures found by Method 1 with different H···A cutoffs and the NCI method with a density-based strength.

Donor	Acceptor	$Dist_{H\cdots A}$ (Å)	R_{min} (Å)	R_{min} – $dist_{H\cdots A}$
Chain A thiolate				
O water	Glu48 O	1.72	3.47	1.75
O water	Glu48 O	1.88	3.47	1.59
O water	Thr42 O	1.92	3.54	1.62
O water	Leu39 O	2.00	3.47	1.47
Arg116 N	O water	2.01	3.62	1.61
O water	Glu48 O	2.13	3.47	1.34
O water	Cys45 S	2.20	3.77	1.57
Thr42 O	Cys45 S	2.29	3.77	1.48
O water	Cys45 S	2.44	3.77	1.33
O water	Cys45 S	2.49	3.77	1.28
Chain B thiolate				
Gln46 N	Thr42 O	2.26	3.62	1.36
Cys45 N	Cys45 S	2.27	3.85	1.58
Arg116 N	Cys45 S	2.30	3.85	1.55
O water	Cys45 S	2.31	3.77	1.46
Thr42 O	Cys45 S	2.33	3.77	1.44
Arg116 N	Cys45 S	2.45	3.85	1.40
Chain A thiol				
Cys45 S	Leu39 O	1.80	3.70	1.90
O water	O water	1.84	3.54	1.70
Thr42 O	O water	1.90	3.54	1.64
Arg116 N	O water	1.98	3.62	1.64
O water	Thr42 O	2.02	3.54	1.52
O water	Leu39 O	2.35	3.47	1.12
Cys45 N	O water	2.51	3.62	1.11
O water	Cys45 S	2.77	3.77	1.00
Chain B thiol				
O water	Thr42 O	1.71	3.54	1.83
Cys45 S	Leu39 O	2.00	3.70	1.70
Cys45 N	O water	2.05	3.62	1.57
Arg116 N	O water	2.24	3.62	1.38
Arg116 N	Cys45 S	2.49	3.85	1.36
Arg116 N	Cys45 S	2.92	3.85	0.93

Table S5. Difference between the H···A distance and the R_{min} distances of the heavy atoms involved in the H-bond. R_{min} = ½($R_{min,D}$ + $R_{min,A}$) with $R_{min,A}$ is the minimum of the Lennard-Jones potential between two atoms A, as implemented in CHARMM.

Supplementary references

1. Hugo M, Turell L, Manta B, Botti H, Monteiro G, Netto LE*, et al.* Thiol and sulfenic acid oxidation of AhpE, the one-cysteine peroxiredoxin from Mycobacterium tuberculosis: kinetics, acidity constants, and conformational dynamics. *Biochemistry* 2009, **48**(40)**:** 9416-9426.

2. Li S, Peterson NA, Kim MY, Kim CY, Hung LW, Yu M*, et al.* Crystal Structure of AhpE from Mycobacterium tuberculosis, a 1-Cys peroxiredoxin. *J. Mol. Biol.* 2005, **346**(4)**:** 1035-1046.

3. Hall A, Parsonage D, Poole LB, Karplus PA. Structural evidence that peroxiredoxin catalytic power is based on transition-state stabilization. *J. Mol. Biol.* 2010, **402**(1)**:** 194-209.

4. Wood ZA, Schroder E, Robin Harris J, Poole LB. Structure, mechanism and regulation of peroxiredoxins. *Trends Biochem. Sci.* 2003, **28**(1)**:** 32-40.

5. Joosten RP, Salzemann J, Bloch V, Stockinger H, Berglund AC, Blanchet C*, et al.* PDB_REDO: automated re-refinement of X-ray structure models in the PDB. *J. Appl. Crystallogr.* 2009, **42**(Pt 3)**:** 376-384.

6. Murshudov GN, Skubak P, Lebedev AA, Pannu NS, Steiner RA, Nicholls RA*, et al.* REFMAC5 for the refinement of macromolecular crystal structures. *Acta Crystallogr. D Biol. Crystallogr.* 2011, **67**(Pt 4)**:** 355-367.

7. Hugo M, Van Laer K, Reyes AM, Vertommen D, Messens J, Radi R*, et al.* Mycothiol/mycoredoxin 1-dependent reduction of the peroxiredoxin

AhpE from Mycobacterium tuberculosis. *J. Biol. Chem.* 2014, **289**(8)**:** 5228-5239.

8. Nakamura T, Kado Y, Yamaguchi T, Matsumura H, Ishikawa K, Inoue T. Crystal structure of peroxiredoxin from Aeropyrum pernix K1 complexed with its substrate, hydrogen peroxide. *J. Biochem.* 2010, **147**(1)**:** 109-115.

9. Collaborative CP. The CCP4 suite: programs for protein crystallography. *Acta Crystallogr. D Biol. Crystallogr.* 1994, **50**(Pt 5)**:** 760.

10. Emsley P, Lohkamp B, Scott WG, Cowtan K. Features and development of Coot. *Acta Crystallogr. D Biol. Crystallogr.* 2010, **66**(Pt 4)**:** 486-501.

11. Chen VB, Arendall WB, 3rd, Headd JJ, Keedy DA, Immormino RM, Kapral GJ*, et al.* MolProbity: all-atom structure validation for macromolecular crystallography. *Acta Crystallogr. D Biol. Crystallogr.* 2010, **66**(Pt 1)**:** 12-21.

12. Johnson ER, Keinan S, Mori-Sanchez P, Contreras-Garcia J, Cohen AJ, Yang W. Revealing noncovalent interactions. *J. Am. Chem. Soc.* **132**, 6498-6506 (2010).

13. Bader RF, Essén H. The characterization of atomic interactions. *J. Chem. Phys.* 1984, **80**(5)**:** 1943-1960.

14. Zhao Y, Truhlar D.G. The M06 suite of density functionals for main group thermochemistry, thermochemical kinetics, noncovalent interactions, excited states, and transition elements: two new

functionals and systematic testing of four M06-class functionals and 12 other functionals. *Theor. Chem. Acc.* 2008, **120**: 215-241.

15. Marenich AV, Cramer CJ, Truhlar DG, Universal Solvation Model Based on Solute Electron Density and on a Continuum Model of the Solvent Defined by the Bulk Dielectric Constant and Atomic Surface Tensions. *J. Phys. Chem. B* 2009, **113**: 6378-6396.

16. Li L, Li C, Zhang Z, Alexov E. On the Dielectric “Constant” of Proteins: Smooth Dielectric Function for Macromolecular Modeling and Its Implementation in DelPhi. *J. Chem. Theory Comput.* 2013, **9**: 2126–2136
